# Improvement of a massage chair (BEG-100) on height growth in children with average: Human subjects research

**DOI:** 10.1097/MD.0000000000020080

**Published:** 2020-05-01

**Authors:** Sun Haeng Lee, Mia Kim, Chuljin Jeon, Soohyun Cho, Min Hyung Choi, Tae Hwan Hwang, Jihong Lee, Gyu Tae Chang, Jin Yong Lee

**Affiliations:** a Department of Clinical Korean Medicine, Graduate School, Kyung Hee University; b Department of Pediatrics of Korean Medicine, Kyung Hee University Korean Medicine Hospital, Kyung Hee University Medical Center; c Department of Cardiovascular and Neurologic Disease (Stroke Center), College of Korean Medicine, Kyung Hee University, Dongdaemun-gu; d Medical R&D Center, Bodyfriend, Gangnam-gu; e Department of Pediatrics of Korean Medicine, Kyung Hee University Hospital at Gangdong, Gangdong-gu, Seoul, Republic of Korea.

**Keywords:** growth, height, massage chair, massage

## Abstract

**Background:**

Studies that examine the growth effects of massage at ages beyond infancy are rare. A recently developed massage chair (BEG-100) targets teenagers who want to increase their growth. The chair can stretch areas around the knee after fixating the knee and ankle areas.

**Methods:**

This is a clinical study that included 38 children aged 11 years whose heights ranged from 145.0 to 155.0 cm. We aim to observe the potential of a 24-week massage for growth promotion. We will also evaluate the changes in height percentile, height, height standard deviation score, bone age, height standard deviation score for bone age, growth rate, predicted height estimated from bone age, seated height-to-standing height ratio, weight, and body mass index. To confirm safety, the associated adverse events will be investigated.

**Objective:**

The aim of this study was to investigate the growth-promoting effect of BEG-100 in children with average height.

**Trial registration:**

KCT0004673 (Clinical Research Information Service).

## Abstract 2

Supplemental Digital Content is available in the text

## 1 Introduction

A massage chair uses mechanical devices such as massage bolls, rollers, and airbags, and automatically massages the whole body.^[1]^ Depending on the development of the technology, the massage sensations elicited by experts, such as tapping, patting, finger pressure, and stretching, could be experienced with the massage chair.^[2]^ Mechanical massage with a massage chair is known to have the effect of reducing muscle strain,^[3]^ improving sleep quality,^[4]^ and recovering mental fatigue and enhancing cognition.^[1]^

If no definite evidence exists on the current or future negative psychological and social concerns associated with short stature, a previous study proposed that the idiopathic short stature should not be treated with growth hormone (grade 2C).^[5]^ This proposal assumes that the psychological and social benefits associated with an additional growth increase are less important than the cost and burden of long-term treatment.

Many alternative treatments have been developed for promoting growth because growth hormone treatment for idiopathic short stature is not recommended. One of these alternative treatments is massage therapy. The growth-promoting effect of massage was mainly reported in premature babies. Massage can lead to 5.1 g of daily weight gain,^[6]^ and is thought to be the result of increased vagal activity and gastric motility in premature babies.^[7]^ Height and thoracic circumference were significantly increased in Korean premature babies who received massage twice a day for 14 days.^[8]^ Although no weight gain was shown in premature boys after massage twice a day for 4 weeks, the quality of growth improved by reducing the adiponectin circulation and body fat accumulation.^[9]^ The massage method affects growth stimulation because the 6-week meridian massage for 15 minutes per day showed higher growth rate than general massage.^[10]^

Studies that examined the growth effects of massage at ages beyond infancy are rare. Therefore, identifying the potential of massage for promoting child growth can provide additional evidence for childhood growth alternative therapy. A recently developed massage chair (BEG-100) can strengthen the stretch around the knee after fixating the knee and ankle areas. Periodic stretching of the soft substrate is known to change substrate rigidity and develop fibrous tissue.^[11]^ Stretching by BEG-100 is expected to have a positive effect on growth.

We prepared a prospective study to investigate the effects of a massage chair on growth by comparing with national standard statistical data. The results will provide information on the potential effects of the massage chair on growth stimulation and the possible adverse events (AEs) associated with the use of the chair in children.

## 2 Methods

### 2.1 Ethics approval

The study will be performed in accordance with the principles of the Declaration of Helsinki and the Ethical Guideline for Clinical Research, and the institutional review board (IRB) of Kyung Hee University Korean Medicine Hospital (KOMCIRB 2019-03-002). The study protocol was registered with the Clinical Research Information Service (KCT0004673).The IRB will check the study protocol at least yearly. Any modifications of the protocol will be approved by IRB before its execution. Korean medicine doctors will explain the study to the children and their guardians and acquire written informed consent. If AEs occur during the study, appropriate medical treatment will be provided until the participant recovers. All the participants’ data will be documented by the code number and participants’ initials, and the documents will be stored in locked file cabinets with controlled access. The study results will be released to the participants, health care professionals, and public via publications.

### 2.2 Study design

This is a prospective single-group study protocol that will include children aged 11 years with average height. It aims to observe the potential of a 24-week massage for growth promotion. The study protocol conforms to the Standard Protocol Items: Recommendations for Interventional Trials.

### 2.3 Participants

**Figure 1:**
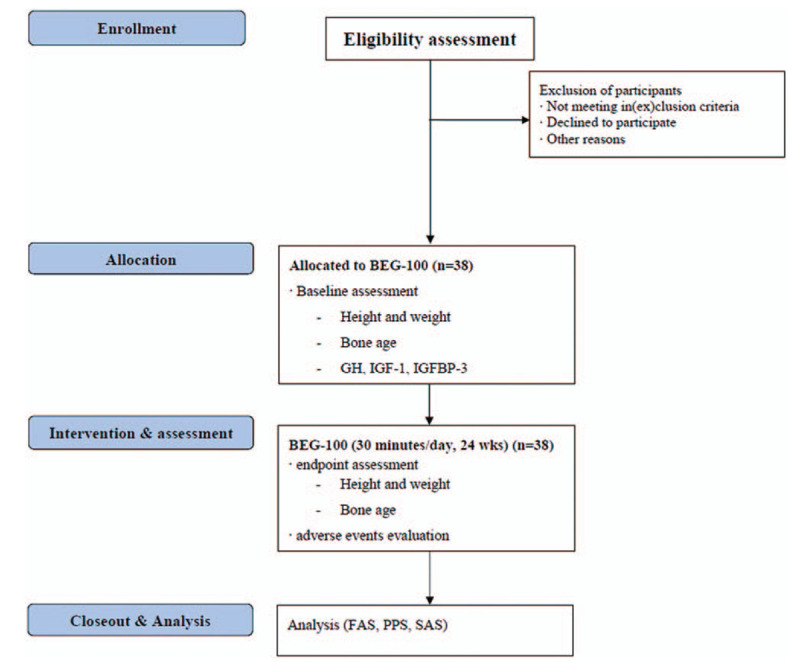
Schematic chart of the study process. FAS = full analysis set, GH = growth hormone, IGF-1 = insulin-like growth factor-1, IGFBP-3 = insulin-like growth factor binding protein-3, PPS = per protocol set, SAS = safety assessment set.

**Figure 2:**
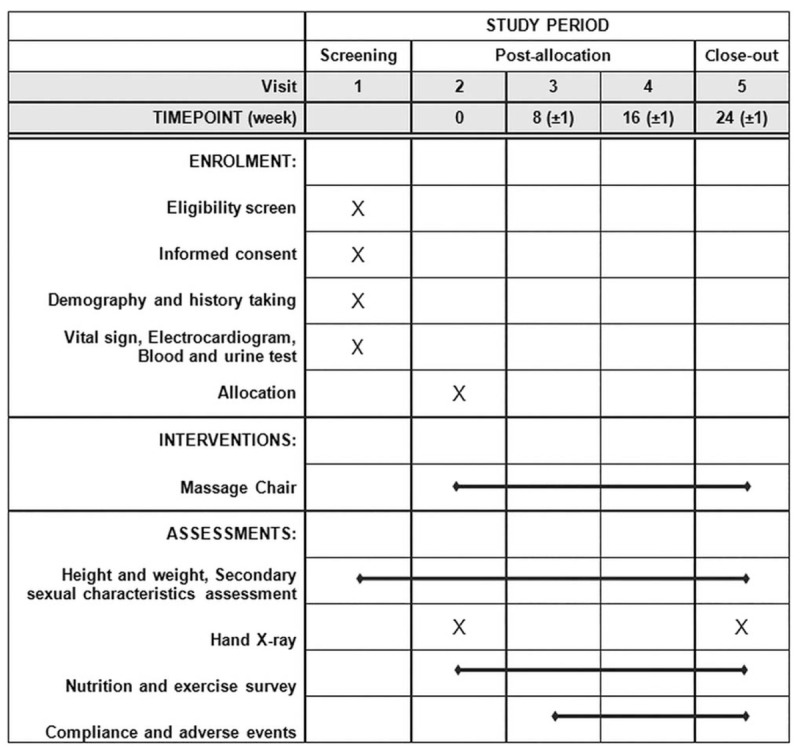
Schedule of enrolment, interventions, and assessments.

Thirty-eight children from Kyung Hee University Medical Center will be recruited through posters and banners. The participants and their guardians will receive research information such as objectives, procedures, and potential benefits and harms through standardized dialogues. Informed consent will be acquired from the children and their guardians ahead of the screening process. The participants will be allowed to withdraw from the study at any time they want. The research procedure will be conducted as shown in Figs. 1 and 2. All the participants will undergo a massage chair treatment at home for 24 weeks. After the screening session, 4 assessment visits will be planned for the children. The guardians will receive text messages as reminders of the scheduled visits.

### 2.4 Sample size

The number of participants was calculated on the basis of comparative studies that verified the effects of massage on growth because previous studies on massage intervention using a massage chair for growth promotion are rare. Thirty children per group were selected for judging the growth effect of massage in previous studies.^[12,13]^ We calculated the appropriate total number of participants to be 38 by considering the 20% dropout rate.

### 2.5 Inclusion criteria

1. Boys and girls aged 11 years whose heights range from 145.0 to 155.0 cm (considering the size of the massage chair)
2. Children who agree to participate in this study and have written informed consent forms signed by their representative.

### 2.6 Exclusion criteria

1. Growth retardation from endocrinopathy (growth hormone deficiency, hypothyroidism, type 1 diabetes, and so on)
2. History of intrauterine growth retardation
3. Diagnosis and treatment of chronic or terminal diseases (hypertension, obesity, hyperlipidemia, diabetes, thyroid disease, cancer, and so on)
4. Diagnosis and treatment of musculoskeletal diseases that make implementation of the massage chair difficult (congenital malformation, scoliosis, osteogenesis imperfecta, musculopathy, and so on)
5. Clinically significant disease or history of diseases of the liver, pancreas, kidney, nervous system, respiratory system, endocrine system, cardiovascular system, urinary system, hemopathy, tumor, or psychopathy, especially a history of severe heart disease or major psychopathy such as mania/hypomania, bipolar disorder, depressive disorder, anxiety disorder, suicidal tendency, schizophrenia, attention deficit hyperactivity disorder, and autism spectrum disorder
6. Treatments for improving height growth such as growth hormone therapy, hormone replacement therapy, medications, health functional foods, and herbal medicines within 4 weeks before the screening assessment
7. Participation in another clinical study within 4 weeks before the screening assessment, or planning to participate in another clinical study during the intervention period
8. Plan to receive treatments for improving height growth within the research period
9. Unsuitability for study participation for reasons other than the aforementioned as judged by the researchers
10. Outside the normal range of the related examination.

### 2.7 Intervention

**Figure 3:**
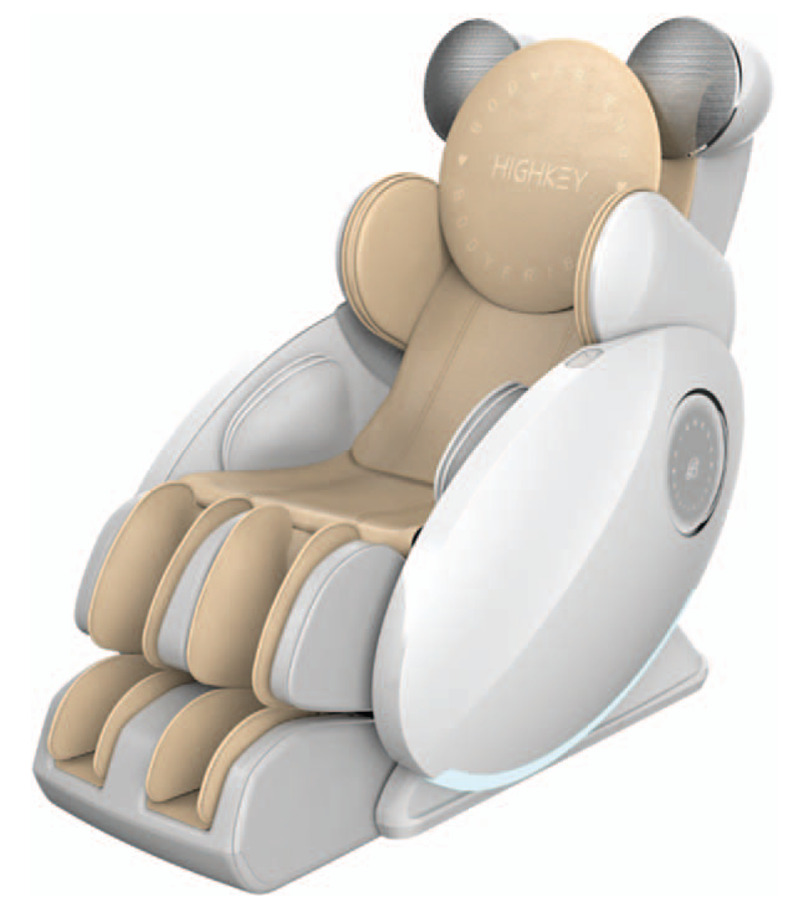
Massage chair (BEG-100).

After the screening, all the appropriate participants will be assigned to the BEG-100 treatment. BEG-100 is a massage chair made by Bodyfriend, Inc.(Seoul, Republic of Korea) to promote height growth (Fig. 3). The manufacturer will deliver BEG-100 to the participants’ home after enrolment and is also responsible for the management and return of the massage chair. All the participants will sit on the chair and apply growth mode (lower body growth for 20 minutes and whole-body growth for 10 minutes) every day for 24 weeks. BEG-100 stimulates the knee growth plate by stretching the knee and lower limbs in the lower body growth mode. It stimulates the vertebral growth plate by stretching the vertebrae and upper body in the upper body growth mode. It stretches the upper and lower body in the whole-body growth mode.

BEG-100 uses a thermal massage ball and thermal airbag for realizing the massage sensation with warm hands. The leg parts of BEG-100 are automatically adjusted to the length of the user's legs ranging from 9 to 20 cm. For the feet, 2 rollers massage the soles and 3 airbags massage the heels.

The participants should not take any other medications, Korean medicines, health functional foods, and treatments that can influence growth. Participants receiving growth-promoting medications, including growth hormone; growth-promoting Korean medicines, including herbal medication tonifying Qi, tonifying blood, or improving digestion; growth-promoting health functional foods, including nutritional supplements; growth-promoting treatments, including leg correction, orthotherapy, physical therapy, rehabilitation, acupuncture, electroacupuncture, pharmacopuncture, bee venom, moxibustion, cupping, cognitive behavioral therapy, and mind-body therapy will be excluded from the study participation.

The participants will stop conducting the intervention with the massage chair and will undergo relevant treatment if AEs associated with BEG-100 occur, such as abrasion, bruise, contusion, muscle and tendon damages, allergic symptoms, burns, and vertebral sprain or fracture. Compliance of device use in the home will be regularly monitored by filling in a self-written tabular form in every visit (Additional file 1.docx, SDC link:).

### 2.8 Outcome measurements

#### 2.8.1 Height

Height will be measured with the participant barefoot, and the value will be rounded off to 2 decimal places. Height will be measured by a trained nurse at the same time of day (±1 hour) to reduce height deviation during the day. Height will be measured with a portable stadiometer (Seca 213, Seca GmbH, Hamburg, Germany) that can measure heights ranging from 20 to 205 cm. Height will be measured from screening to close-out.

Height percentile and standard deviation score (SDS) will be based on the 2017 Korean growth chart.^[14]^ Values outside 3 standard deviations from the median of the height were removed to avoid biased calculation. The height percentile was smoothed by Loess regression (Proc Loess in SAS 9.4) for removing fluctuation associated with age or height. The values were converted to normal distributions by applying the Box-Cox transformation.

Growth rate will be calculated by dividing the height difference by the number of days required. The seated height-to-standing height ratio will be determined by measuring height in the seated position with Seca 213.

#### 2.8.2 Bone age

On hand radiography at visits 2 and 5, bone age will be computed using the Tanner-Whitehouse (TW) method. The maturity of the hand ossification centers will be assessed as a radius-ulnar-short bone (RUS) score from 0 to 1000 as compared with the bone age chart according to sex and age.^[15]^ Two-year-old boys have a RUS score of 42, and 2-year-old girls have a RUS score of 126. The RUS score of 1000 is computed for 16.5-year-old boys and 15.0-year-old girls. The TW3 RUS score was based on the American, Argentine, Belgian, British, Italian, Japanese, and Spanish surveys between 1960 and 1995. The TW3 RUS score will be calculated by researchers who have been trained for > 3 months before assessment.

Predicted adult height will be calculated from the measured height and RUS score using the TW3 equations, which were developed according to boys, girls before menarche, and girls after menarche. The height SDS for bone age will be determined according to the 2017 Korean growth chart.

#### 2.8.3 Weight

Weight will be measured in light clothing and the value will be rounded off to 2 decimal places. Weight will be measured by a trained nurse using a load cell scale (Jenix DS-103 M) that can measure weights ranging from 10 to 200 kg. Weight will be measured from screening to close-out. Body mass index (BMI) will be calculated by dividing the weight by the square of the height.

#### 2.8.4 AEs

To assess overall clinical safety, the researchers will collect all voluntarily reported AEs during each visit and record the name, date, severity, course, seriousness, causality, and treatment performed for the AE in the case report form. The participants and their guardians will be asked, “Has there been any AE since the last visit?” When the child does not report any AE, the severity is considered minor or nonexistent. If the reported AEs are not sufficient for the participant to drop out from the study, the severity is considered moderate. When the child has to withdraw from the study to undergo treatment for the AE, the severity is considered severe.

### 2.9 Statistical analyses

Outcomes will be analyzed with a full analysis set (FAS) and per protocol set (PPS). The FAS targets all participants whose heights can be measured after starting the intervention and have no major violation of inclusion criteria. PPS targets all the participants who have been treated with the massage chair for >118 days (70% of the total 168 days). The safety analysis targets all participants with available safety data obtained by phone or visit.

Continuous data will be arranged as representative values such as mean and standard deviation. Height percentile, height SDS, predicted adult height, seated height-to-standing height ratio, and BMI will be tested by using the paired *t* test or Wilcoxon signed-rank test. The final height and weight will be measured by using a 1-sample *t* test or Wilcoxon signed rank-test from the parameters after 24 weeks of initial percentile. The final bone age will be tested by using a 1-sample *t* test or Wilcoxon signed rank-test, from the age after 0.5 years of the initial bone age. AEs will be described as frequency, percentage, and 95% confidence interval for the occurrence rate in all the participants. The difference in the incidence of AEs between before and after intervention will be tested by using a *χ*^2^ test or Fisher exact test. A secondary analysis will be conducted by grouping according to boys, girls before menarche, and girls after menarche. If necessary, analysis of covariance will be used.

The significance level is 0.05 for all the statistical tests, and 2-tailed tests will be performed. Missing values will be imputed using the last-observation-carried-forward method.

### 2.10 Data monitoring

The data monitoring committee is composed of three Korean medicine doctors employed in Kyung Hee University Korean Medicine Hospital but who have no participation in this study. They will conduct monthly monitoring of document reporting, protocol violation, and AEs during the study. Any interim analyses and guidelines for discontinuation will follow the standard operating procedure of the Korean Medicine Clinical Trial Center of Kyung Hee University Korean Medicine Hospital. Issues found will be discussed with the principal investigator.

## 3 Discussion

Massage had some effects on growth in randomized controlled trials (RCTs) or quasi-RCTs.^[16]^ It was used alone or with swimming, oral administration, plaster therapy, or auricular acupuncture. The most frequently used massage method was spine pinching, which promotes gastric fluid secretion, gastrointestinal peristalsis, and digestive ability for carbohydrates and proteins. However, previously studied growth-promoting massages have individual differences in effect depending on the skill of the practitioner, due to the nature of manual therapy. This is the first study to verify the effect of standardized massage using massage chair on growth by stimulating the spine and knees with fixed strength and frequency.

BEG-100 targets children whose heights range from 130 to 170 cm, but it is most appropriately manufactured for a 150-cm height. We determined to enroll 11-year-old Korean children because the average heights of 11-year-old Korean boys range from 144.7 to 150.8 cm and those of 11-year-old Korean girls range from 145.8 to 151.3 cm. The intervention period was set to be 24 weeks, which is the most frequent intervention period in previous studies that reported the effect of massage on growth. Standing and seated heights will be measured to see changes in height, height percentile, height SDS, growth rate, and seated height-to-standing height ratio. Hand radiography will be performed to identify changes in bone age, predicted adult height, and height SDS for bone age. Through weight measurements, we will confirm changes in weight, weight percentile, and BMI. The safety of BEG-100 will be investigated through a comparison of voluntarily reported AEs before and after the intervention.

The study protocol has a few limitations. First, a comparison group was not included. Although the data from the intervention group will be compared with national standard statistical data, the growth effect of the massage chair cannot be proved because of the absence of a local comparison group. This study is only a pilot study for future randomized controlled trials. Second, the frequency of the intervention is difficult to ascertain. Massage using BEG-100 will be performed in the participants’ homes, and the frequency of the intervention will be confirmed by the participants’ reports. The participants might report more or less frequency about the intervention, but this is unlikely because of the absence of a related reward or penalty. Third, although nutrition balance and exercise time will be checked in every visit after the BEG-100 intervention, other confounding factors regarding growth such as stress and sleep may influence the results.

Massage stimulates skin sensory receptors, subcutaneous tissues, blood, lymph, and tissue fluid; therefore, increasing local microcirculation and parasympathetic nervous system.^[17]^ It also increases food absorption and basal metabolism through enhanced vagal activity, gastric motility, and insulin and insulin-like growth factor-1 levels.^[18]^ Spine massage promotes local skin protein expression, thereby regulating the local immune function.^[19]^ Moderate pressing stroke, and flexion and extension of the extremities increased bone mineral density and bone growth.^[7]^ However, these growth effects of massage have been mainly demonstrated in premature babies. Our research will provide information on the potential effects and safety of massage using a massage chair for growth in teenagers.

## Acknowledgments

The authors thank Editage (www.editage.co.kr) for English language editing.

## Author contributions

**Conceptualization:** Chuljin Jeon.

**Data curation:** Tae Hwan Hwang.

**Funding acquisition:** Jin Yong Lee.

**Investigation:** Min Hyung Choi.

**Methodology:** Gyu Tae Chang.

**Project administration:** Sun Haeng Lee.

**Resources:** Mia Kim.

**Software:** Sun Haeng Lee.

**Supervision:** Soohyun Cho.

**Validation:** Jihong Lee.

**Visualization:** Sun Haeng Lee.

**Writing – original draft:** Sun Haeng Lee.

**Writing – review & editing:** Mia Kim, Chuljin Jeon, Soohyun Cho, Min Hyung Choi, Tae Hwan Hwang, Jihong Lee, Gyu Tae Chang, Jin Yong Lee.

## Supplementary Material

Supplemental Digital Content
