# Supplementary material for: Improvement of a massage chair (BEG-100) on height growth in children with average: Human subjects research
Source: Medicine (Baltimore). 2020 May 1;99(18):e20080. doi: 10.1097/MD.0000000000020080 (PMC7440093; doi:10.1097/MD.0000000000020080)
Supplement: Supplemental Digital Content [file medi-99-e20080-s001.docx]

**Additional file 1. Protocol version**

| Version | Date | Action |
| --- | --- | --- |
| 1.0 | 2018-Dec-29 | Protocol draft development |
| 1.1 | 2019-Jan-05 | Minor revision |
| 2.0 | 2019-Jan-22 | Statistical revision |
| 2.1 | 2019-Mar-14 | Minor revision |
| 3.0 | 2019-Apr-19 | Checkup frequency revision |
| 4.0 | 2019-May-17 | Revision following institutional review board’s comments |
| 4.1 | 2019-Aug-06 | Minor revision |
| 5.0 | 2019-Oct-10 | Revision for single group study |
| 5.1 | 2019-Nov-12 | Minor revision |
